# GNP-GAPDH_1-22_ nanovaccines prevent neonatal listeriosis by blocking microglial apoptosis and bacterial dissemination

**DOI:** 10.18632/oncotarget.19405

**Published:** 2017-07-20

**Authors:** Ricardo Calderon-Gonzalez, Elisabet Frande-Cabanes, Hector Teran-Navarro, José María Marimon, Javier Freire, David Salcines-Cuevas, M. Carmen Fariñas, Claudia onzalez-Rico, Marco Marradi, Isabel Garcia, Mirian Alkorta-Gurrutxaga, Aida San Nicolas-Gomez, Ana Castañeda-Sampedro, Sonsoles Yañez-Diaz, Soledad Penades, Carmen Punzon, Javier Gomez-Roman, Fernando Rivera, Manuel Fresno, Carmen Alvarez-Dominguez

**Affiliations:** ^1^ Grupo de Nanovacunas y vacunas celulares basadas en Listeria y sus aplicaciones en biomedicina, Instituto de Investigación Marqués de Valdecilla, Santander, Spain; ^2^ Servicio de Microbiología, Instituto de Investigación Sanitaria Biodonostia, Hospital Universitario Donostia, San Sebastián, Gipuzkoa y CIBER de Enfermedades Respiratorias (CIBERES), Madrid, Spain; ^3^ Servicio de Anatomía Patológica, Hospital Universitario Marqués de Valdecilla, Santander, Spain; ^4^ Seccion de Enfermedades Infecciosas, Hospital Universitario Marqués de Valdecilla, Santander, Spain; ^5^ CIC biomaGUNE, and Biomedical Research Networking Center in Bioengineering, Biomaterials and Nanomedicine (CIBER-BBN), Paseo Miramón 182, Donostia-San Sebastián, Spain; ^6^ Servicio de Dermatología, Hospital Universitario Marqués de Valdecilla, Santander, Spain; ^7^ Diomune S.L. Parque Científico de Madrid, Madrid, Spain; Centro de Biología Molecular Severo Ochoa, Universidad Autónoma de Madrid, Madrid, Spain; ^8^ Servicio de Oncología Médica, Hospital Universitario Marqués de Valdecilla, Santander, Spain

**Keywords:** neonatal listeriosis, microglia, apoptosis, tumor necrosis factor signaling, nanovaccines, Immunology and Microbiology Section, Immune response, Immunity

## Abstract

Clinical cases of neonatal listeriosis are associated with brain disease and fetal loss due to complications in early or late pregnancy, which suggests that microglial function is altered. This is believed to be the first study to link microglial apoptosis with neonatal listeriosis and listeriosis-associated brain disease, and to propose a new nanovaccine formulation that reverses all effects of listeriosis and confers *Listeria monocytogenes* (LM)-specific immunity. We examined clinical cases of neonatal listeriosis in 2013–2015 and defined two useful prognostic immune biomarkers to design listeriosis vaccines: high anti-GAPDH_1-22_ titres and tumor necrosis factor (TNF)/interleukin (IL)-6 ratios. Therefore, we developed a nanovaccine with gold glyco-nanoparticles conjugated to LM peptide 1-22 of GAPDH (Lmo2459), GNP-GAPDH_1-22_ nanovaccinesformulated with a pro-inflammatory Toll-like receptor 2/4-targeted adjuvant. Neonates born to non-vaccinated pregnant mice with listeriosis, showed brain and vascular diseases and significant microglial dysfunction by induction of TNF-α-mediated apoptosis. This programmed TNF-mediated suicide explains LM dissemination in brains and livers and blocks production of early pro-inflammatory cytokines such as IL-1β and interferon-α/β. In contrast, neonates born to GNP-GAPDH_1–22_-vaccinated mothers before LM infection, did not develop listeriosis or brain diseases and had functional microglia. In nanovaccinated mothers, immune responses shifted towards Th1/IL-12 pro-inflammatory cytokine profiles and high production of anti-GAPDH_1–22_ antibodies, suggesting good induction of LM-specific memory.

## BACKGROUND

Cerebral listeriosis caused by the human pathogen *Listeria monocytogenes* (LM) constitutes a severe disease in infants and elderly people, with a mortality rate of 30% [1]. LM targets the central nervous system (CNS) and foetus, and the most common listeriosis-related diseases are meningitis, meningoencephalitis, brain abscesses, foetal malformations and septicaemia [2–4]. Since 2008, the cases of listeriosis have increased in the European Union, but especially in Spain, where listeriosis cases reported between 2008 and 2014 are 1.15 cases per 100,000 inhabitants [5–7]. However, the landscape can be worse as listeriosis has only been listed as a notifiable disease in Spain since March 2015 [8], and therefore, annual incidences may be higher. Despite the amassing epidemiological data on listeriosis, immunological biomarkers were not investigated till recently [9]. Moreover, a vaccine that prevents neonatal listeriosis and avoid or at least diminish brain diseases and foetal injury is not yet available but necessary. Our group has been preparing vaccines for systemic listeriosis in experimental models with good success. Dendritic cells (DCs) loaded with two LM immunodominant peptides, peptide 91-99 of listeriolysin O virulence factor, LLO_91-99_ and peptide 1-22 of glyceraldehyde-3-phosphate dehydrogenase (Lmo2459), GAPDH_1-22_ [10–12] or phagosomes obtained from macrophages and containing live wild type LM (LM^WT^) [13], prevented systemic listeriosis in mice. However, none of these vaccine vectors appeared suitable for neonatal listeriosis, because phagosomes containing live LM^WT^, supposed a risk for pregnant mothers, as they may develop listeriosis. Moreover, DCs poorly crossed the placenta and blood-brain barriers as a putative strategy to avoid injuring the foetus or causing neuroinflammation [14, 15].

Microglia (MG) are resident macrophages that survey the brain parenchyma to repair any damage, control pathogen invasion and prevent neuron injury. Therefore, they are responsible for controlling LM infection and potential target cells to combat listeriosis in the brain of humans, mice or ruminants [16–19].

Similar to macrophages, MG are divided into M1 and M2 subsets depending on their pro- or anti-inflammatory cytokine pattern and similar to macrophages [20–24]. In this regard, pro-inflammatory M1 macrophages or MG are microbicidal phagocytes and antigen-presenting cells (APC) that produce soluble and neurotoxic inflammatory mediators such as tumour-necrosis factor (TNF)-α, IL-1β, IL-12, NO or type I interferon (IFN-α/β) [20]. However, continuous activation of M1 subset of MG might disrupt the delicate balance in the CNS and have a negative effect on neuronal survival [20, 24]. Upon microbial infection, M1 macrophages also induce programmed cell death, such as necrotic-programmed cell death, TNF-mediated apoptosis or pyroptosis [25–28]. In fact, apoptosis may be understood as a host mechanism to control infection [28] or a virulence process to spread the pathogen [29]. M2 subset of MG parallel the alternative activation pattern of macrophages and showed high levels of IL-6 and IL-10; activation of pro-inflammatory negative regulators such as suppressor of cytokine signalling (Socs3); low phagocytic functions; promotion of immunoregulation and neuroprotection; and presented low production of reactive oxygen species [22–24].

The aims of this study were as follows. (1) To examine clinical cases of listeriosis to search for immune biomarkers to prepare safe nanovaccines. (2) To establish a neonatal cerebral listeriosis model in mice to investigate brain disease, biomarkers detected in human listeriosis cases, microglia function and apoptosis and bacterial virulence factors. (3) To test gold glyconanoparticle (GNP) vaccine formulations with adjuvants, that help to prevent listeriosis and brain-associated morbidities, including microglial dysfunction.

## RESULTS

### Biomarkers of poor prognosis in neonatal listeriosis

**Figure 1:**
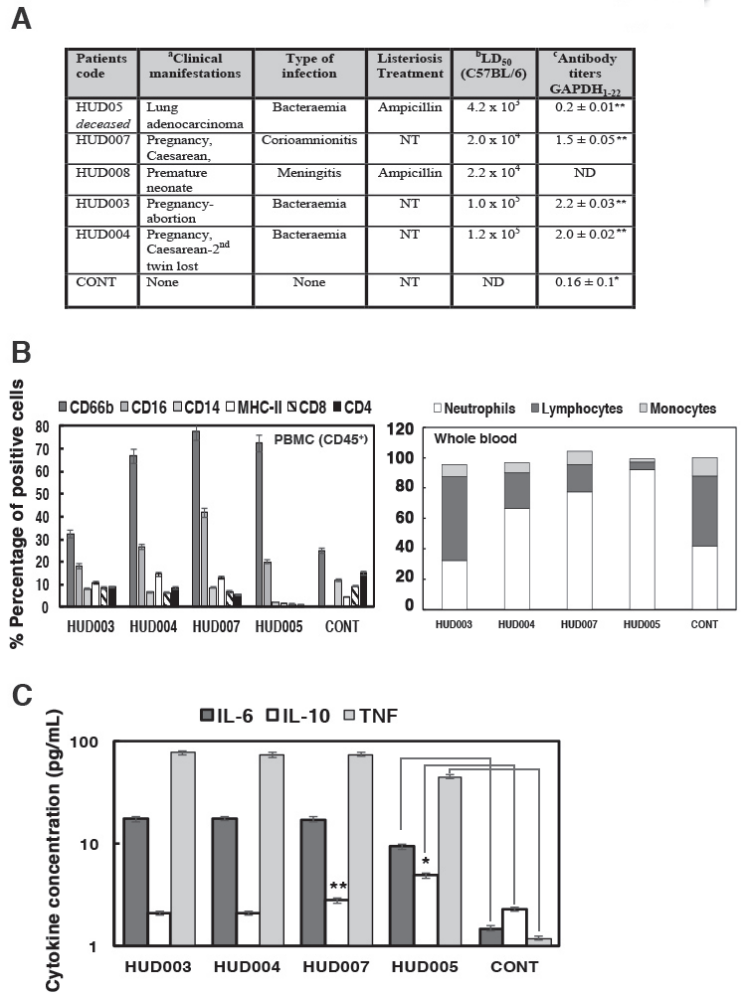
Clinical manifestations and biomarkers of patients with neonatal listeriosis **A**. Clinical manifestations and treatments of patients with listeriosis during 2013-2015. ^a^Patients identified by internal codes. HUD: Hospital Universitario de Donostia (Gipuzkoa). NT, no treatment. ^b^Virulence of clinical bacterial isolates (LD_50_). Genotyping and serotyping were measured via PCR multiplex and sequence types (ST) were determined using MLST [5]. Spleens of C57BL/6 mice infected with 5000 CFU/mL of LM isolates from patients were recovered after 72 h and examined for CFU/mL. We recovered 250 CFU/mL in the spleens of mice infected with 10403S LM strain and 240 CFU/mL in mice infected with EGD strain (these were controls of virulence). ^c^Sera of listeriosis patients were examined for peptide ELISA using GAPDH_1-22_ peptides or LLO_189-201_ peptides. Results are presented as OD and mean ± SD values of triplicate experiments. Anti-LLO_189-201_ titers were <0.2 ± 0.03 OD units and therefore non-significant, except for patient HUD003 with 0.4 ± 0.05 OD units. Negative controls correspond to levels observed in sera of healthy donors (controls). **B**. PBMCs (CD45^+^) of patients with listeriosis were stained with different antibodies to label cell surface markers. Cells were analysed by FACS. Results are expressed as mean ± SD percentages of positive cells. Controls correspond to the percentages obtained in healthy donors (controls). **C**. Levels of pro-inflammatory cytokines in sera of listeriosis patients analysed by flow cytometry. Results are expressed as the mean ± SD concentration (pg/mL) of three independent experiments. Controls correspond to levels detected in sera of healthy donors (control). ANOVA was applied to cytokine results. *P ≤ 0.05, **P ≤ 0.01.

To develop a clinical vaccine that prevented neonatal listeriosis, we explored cases of neonatal listeriosis in 2013-2015 to obtain information about common diseases and immune markers of poor prognosis in the two health institutions participating in this study, Hospital Universitario Donostia (HUD) and Hospital Universitario Marqués de Valdecilla (HUMV). From a total of 13 listeriosis cases in HUD, three cases corresponded to neonatal listeriosis due to complications during pregnancy, a case in the first trimester causing an abortion (HUD003 patient in Figure 1A) and two cases at late gestational period, one neonate born with severe meningitis and treated for a month with ampicillin (HUD008 patient is the neonate of HUD007 patient) and a patient with caesarean of twins and first twin born healthy, while the second twin died (HUD004 patients). Therefore, neonatal listeriosis cases presented with a 50% mortality rate. While neonates with listeriosis presented severe brain diseases, mothers only presented small diarrhea or fever. In the same period, we also had an adult patient with listeriosis who died from lung adenocarcinoma and bacteraemia (HUD005), and served as a positive control for poor listeriosis prognosis (Figure 1A). High virulence of clinical isolates from listeriosis patients appeared to correlate with prognosis, as clinical isolates from mothers with neonatal listeriosis (HUD003, HUD004, HUD007 and HUD008) had 2-10 times higher virulence (LD_50_) than standard LM strains such as 10403S in C57BL/6 congenic mice; a model that is highly resistant to LM infection. A clinical isolate from the deceased patient (HUD005) had 200-fold higher LD_50_. Mice infected with standard 10403S LM strain served as controls of virulence (data in Figure legend). Other reported biomarkers of poor prognosis in sera of adult listeriosis patients [9] are: (1) low levels of antibodies against immunogenic LM virulence factors such as GAPDH (GAPDH_1-22_ epitope) or listeriolysin O (LLO_189-201_ epitope). Negative controls correspond to healthy donors where antibody titres were considered basal levels. (2) The imbalance in the cell populations among peripheral blood mononuclear cells (PBMCs) or whole blood cells (Figure 1B), such as high percentage of granulocytes (CD66b^+^) and low percentage of monocytes (CD14^+^) or CD8^+^ or CD4^+^ T lymphocytes (patient HUD005, Figure 1B). Patients with neonatal listeriosis presented with high titres of anti-GAPDH_1-22_ and undetectable anti-LLO_189-201_ antibodies, normal percentages of monocytes and CD8^+^ T cells, and higher numbers of mature and immature granulocytes as a result of bacterial infection (Figure 1B). A predominant Th2 cytokine profile with low Th1-Th17/IL-6 ratios, is also a biomarker of poor prognosis in adult listeriosis because it favours bacterial growth [1, 3, 4, 9]. We observed that neonatal listeriosis patients also presented low TNF-α/IL-6 ratios, indicating predominant Th2 immune responses (Figure 1C). Controls of cytokine measurements correspond to healthy donors (controls in Figure 1C). Patients with neonatal listeriosis presented some biomarkers of poor prognosis, such as hypervirulent LM strains and low ratios of TNF-α/IL-6, as well as some biomarkers of better prognosis, such as high titres of anti-GAPDH_1-22_ antibodies and normal percentages of monocytes and CD8^+^ T cells. This indicates that efforts should be concentrated on shifting from Th2 to Th1 cytokine profiles to decrease the risk of listeriosis in pregnant women.

### Brain disease in neonatal listeriosis depended on *actA* and *hly* genes, while microglial targeting depended on *actA* gene

**Figure 2:**
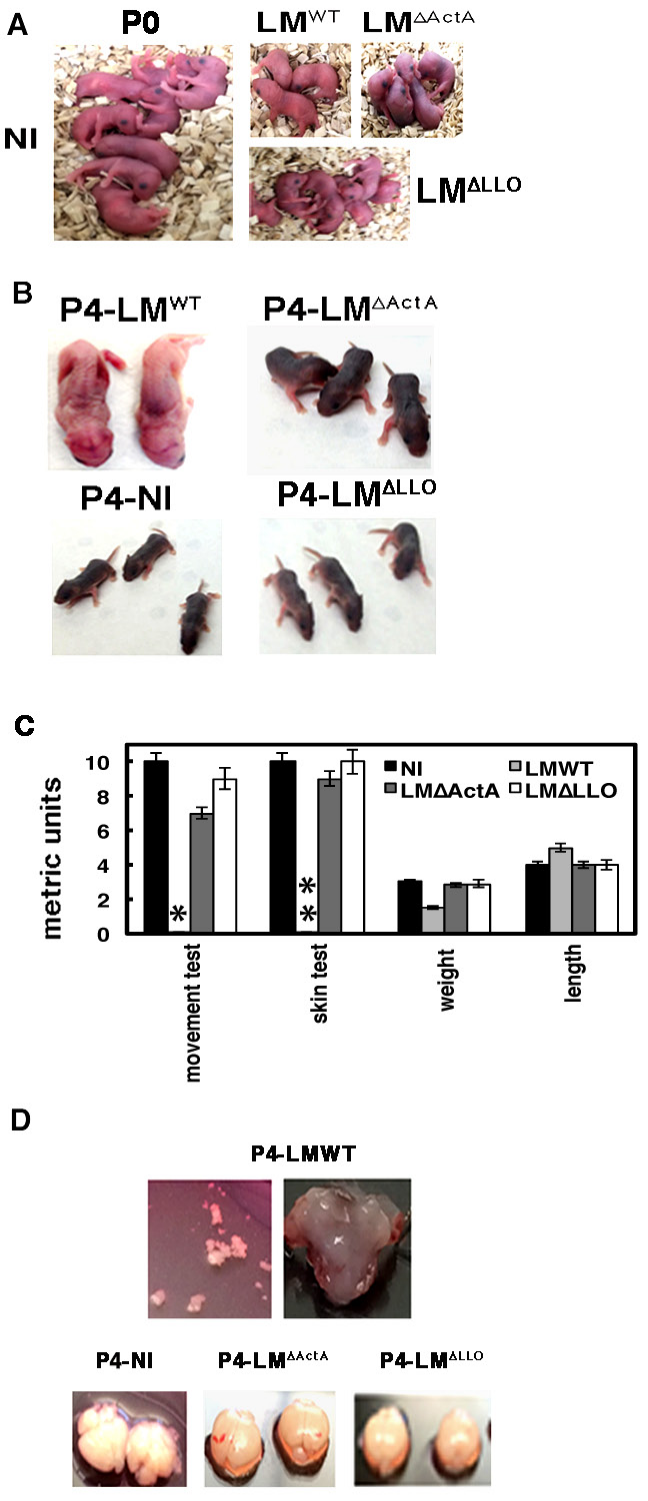
Brain disease in neonatal listeriosis depended on ***actA*** and ***hly*** genes of the pathogen. **A**. P0 neonates born to GFP-LM^WT^-, GFP-LM^∆LLO^- and GFP-LM^∆ActA^-infected or NI mothers. **B**. Comparison of P4 neonates from Panel A for physical and skin appearance and motility. **C**. Clinical tests performed in P4 neonates: movement test, skin test, length, weight and metrics. Results are the mean ± SD of three different experiments. *Bars correspond to 0.1 ± 0.01 cm. **Bars correspond to 0.5-1 melanoblasts/mm of skin. **D**. Brains isolated from P4 neonates of LM^WT^-, LM^∆LLO^- and LM^∆ActA^-infected or NI mothers. Upper images show the fragility of brain covering in P4 neonates born to LM^WT^-infected mothers.

We developed a mouse model of neonatal listeriosis to evaluate brain disease and biomarkers detected in humans, exploring also putative bacterial virulence factors. Adult listeriosis has a 30% mortality rate, and in neonatal listeriosis, meningitis, encephalitis and cerebritis are the main brain diseases, which have higher mortality rates [9, 30–36]. We used five pregnant C57BL/6 female mice at 16 days of embryonic gestation (E16) (n = 5), as a model of neonatal listeriosis and challenged them intravenously (*i.v*) or not (not infected; NI) with 3000 CFU/mL of wild-type GFP-LM^WT^ (LM^WT^), GFP-LM^∆LLO^ (LM^∆LLO^) or GFP-LM^∆ActA^ (LM^∆ActA^) mutants that encode LLO and ActA virulence factors, respectively. Both virulence factors are involved in microglial infection *in vitro* [19]. At E20 we observed 3 ± 0.5 pups born to LM^WT^-infected mothers, 4 ± 0.5 pups born to LM^∆ActA^-infected mothers, 8 ± 0.1 pups born to LM^∆LLO^-infected mothers and 9 ± 0.2 pups born to NI mothers (Figure 2A, images correspond to a representative experiment out of three independent ones, *P* ≤ 0.05). This indicated a 66% mortality rate, which was higher than in humans because we did not treat infected mice with antibiotics. Only two P4 neonates born to LM^WT^-infected mothers survived (Figure 2B), while all other pups born to either LM^∆LLO^- or LM^∆ActA^-infected or NI mothers were alive (Figure 2B). We chose P4 neonates as they corresponded to age 1 month in human neonates; the period with antibiotic treatment in listeriosis and hair development in mice. These results suggest involvement of *actA* gene in foetal wastage as reported previously [30–32], which explains the lower number of neonates in mothers infected with LM^∆ActA^ mutants. The lack of skin pigmentation was noticed only in P4 neonates born to LM^WT^-infected mothers. We investigated all P4 neonates for clinical data, weight, length, coordination movement tests and general observations with a magnifying lens before they were killed. Clinical data were normal in P4 neonates born to NI or LM^∆LLO^- or LM^∆ActA^-infected mothers (Figure 2C), although movement test performance was 30% reduced in the latter. P4 neonates born to LM^WT^-infected mothers showed 50% weight loss, no ability to move in the metric paper test, and a low number of black hair bulbs per millimeter of skin (0.1 ± 0.01) with a wrinkled skin appearance (Figure 2B, 2C). The lack of coordinated movements and melanoblast migration to hair bulbs in P4 neonates born to LM^WT^-infected mothers, confirmed severe CNS retardation after the E16 embryonic stage [33, 34]. We also detected cerebritis or enlarged heads in P4 neonates born to LM^WT^-infected mothers (Figure 2B). We noticed softer cranial covering, lack of brain integrity and a significant reduction of cerebral blood vessels in P4 neonates born to LM^WT^-infected mothers (Figure 2D), which might explain their difficulties in moving and developmental delay. Cell counts in the brain indicated a severe 44% loss of cellularity in P4 neonates born to LM^WT^-infected mothers, compared to normal cellularity in P4 neonates born to LM^∆LLO^- or LM^∆ActA^-infected mothers (3.96 × 10^6^ compared with 9 × 10^6^ cells, Figure 2C). We conclude that the main brain disease, cerebritis, reduction of cerebral blood vessels and cellularity, lack of brain integrity, and melanoblast migration to the hair bulbs seemed to depend on *actA* and *hly* genes.

**Figure 3:**
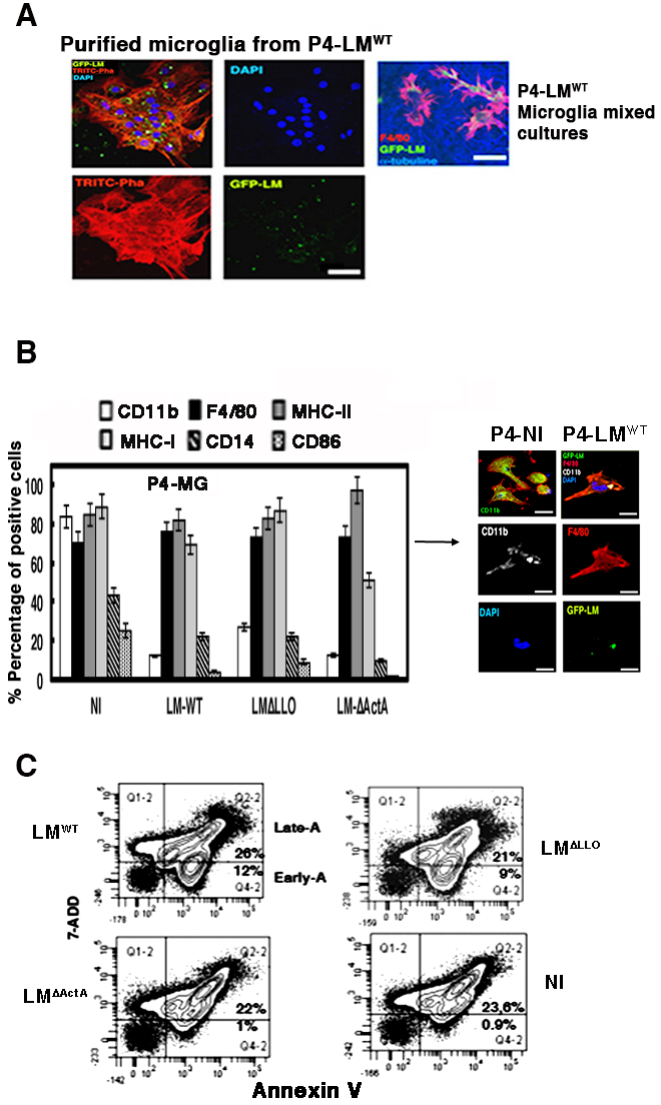
Neonatal listeriosis targets microglia and induces apoptosis **A**. Confocal images of purified microglia containing GFP-LM^WT^ showing that this pathogen has tropism for microglia. Left four images correspond to confocal microscopy images of microglia of neonates born to GFP-LM^WT^-infected mothers. Images of purified microglia containing GFP-LM^WT^ showing that this pathogen has tropism for microglia. Actin filaments are labelled with TRITC-phalloidin and nuclei with 4’,6-diamidino-2-phenylindole (DAPI; blue fluorescence) (isolated microglia from P4-LM^WT^ images). Right images labelled as P4-LM^WT^ correspond to mixed microglial cultures showing that GFP-LM^WT^ (green channel) invaded microglial cells labelled with F4/80-PE antibody (red channel), while surrounding neurons labelled with anti-tubulin β3 antibody (blue channel) did not show intracellular bacteria. Bars, 10 μm. **B**. Left plot represents the cell surface markers analysed by FACS from isolated microglia of P4 neonates born to NI mothers, and infected *in vitro* with LM^WT^, LM^∆LLO^ or LM^∆ActA^ at MOI of 10:1 (bacteria: cell). Results are expressed as the mean ± SD of percentages of positive cells (*P* ≤ 0.05). Right images represent isolated microglia of P4 neonates born to GFP-LM^WT^-infected (P4-LM^WT^) or NI (P4-NI) mothers and analysed by confocal microscopy using different markers, CD11b-APC (LM^WT^) or CD11b-FITC (NI), F4/80-PE and DAPI to stain nuclei. Co-localization of CD11b, GFP-LM^WT^ and F4/80-PE fluorescence was detected as light yellow fluorescence (P4-LM^WT^ images). **C**. Microglia as in Panel A were infected with LM^WT^, LM^∆LLO^ or LM^∆ActA^ for 20 min, washed and stained for annexin V-APC and 7-ADD. Results are expressed as the percentages of late apoptotic cells (Late-A, Q2-2 area corresponding to 7-ADD^+^ annexin V^+^ cells) and the percentages of early apoptotic cells (Early-A, Q4-2 area corresponding to annexin V^+^ 7-ADD^−^ cells) (mean ± SD) (*P* ≤ 0.05).

We investigated LM target cells in the brains of our neonatal listeriosis model, establishing mixed microglial cultures from the hippocampus (95% neurons and 2% microglial cells) of P4 neonates born to LM^WT^-infected mothers. We confirmed a large amount of GFP-LM^WT^ (green fluorescence) localized almost exclusively in microglial cells labelled with the macrophage marker F4/80 (red fluorescence), while neurons stained for α-tubulin were not infected (blue fluorescence in microglial mixed cultures; Figure 3A). We verified LM viability in microglia of P4 neonates born to LM^WT^-infected mothers and detected 6200 CFU/mL, which was 20%-25% of total CFU of pregnant mothers (Table 1). We also followed listeriosis in pregnant mothers infected with LM^WT^ and confirmed normal ability to clear LM infection. CFU/mg in the spleen were lower than in the liver and similar to those in non-pregnant female mice and LM^WT^-infected mothers (Table 1). In mice infected with 10^3^-fold less virulent LM^∆ActA^ strain [31, 32], 3.5 CFU/mg were detected in microglial cells. We also detected significant numbers of bacteria in the liver of LM^∆ActA^-infected mothers (340 CFU) and 10-fold fewer bacteria in the spleen (Table 1). Neonates born to LM^∆LLO^-infected mothers showed no detectable bacteria in microglia, liver or spleen, which suggested these mutants did not cross the placental or brain barriers, while LM^∆ActA^ mutants did (Table 1). Therefore, *actA* but not *hly* gene participated in microglial targeting.

**Table 1:** Listeriosis profile in neonates, pregnant and non-pregnant female mice

Organs (mice type)^a^	CFU/mL^b^
^b^Microglia (LM^WT^)	6.2 × 10^3^ ± 0.1
^b^Microglia (LM^∆LLO^)	0 ± 0
^b^Microglia (LM^∆ActA^)	3.5 ± 0.1*
Liver (NP-LM^WT^)	9.9 × 10^3^ ± 0.2
Liver (Mo-LM^WT^)	2.48 × 10^4^ ± 0.2
Liver (Mo-LM^∆LLO^)	0 ± 0.1
Liver (Mo-LM^∆ActA^)	3.4 × 10^2^ ± 0.1
Spleen (NP-LM^WT^)	8.5 × 10^1^ ± 0.1
Spleen (Mo-LM^WT^)	6.5 × 10^1^ ± 0.1
Spleen (Mo-LM^∆LLO^)	0 ± 0
Spleen (Mo-LM^∆ActA^)	4.3 ± 0.1*

^a^P4 neonates born to LM^WT^-, LM^∆LLO^- or LM^∆ActA^-infected mothers were killed and microglia were isolated from brains, homogenized and plated on brain–heart infusion (BHI) agar. Mothers were also killed (Mo-LM^WT^) as well as non-pregnant female mice infected with LM^WT^ (NP-LM^WT^), and livers and spleens were homogenized and plated on BHI agar. Results are expressed as CFU/mL. ^b^Results are expressed as CFU/mL. (*P* < 0.5), (*P < 0.1)

### Neonatal listeriosis induces an apoptotic cell surface phenotype in microglia

LM infection of phagocytes induces different types of apoptosis or necrosis and significant cell surface remodeling [27–29]. We investigated cell surface changes in the microglia of P4 neonates born to LM^WT^-infected mothers and focused on apoptotic markers. We detected lower percentages of CD11b^+^ (8.2% of positive cells), CD14^+^ (3.9% positive cells), CD11c^+^ (6.3% positive cells), CD86^+^ (2% of positive cells) and MHC-I^+^ (from 67%-90% of positive cells), and normal percentages of MHC-II^+^ (43.1% of total positive cells) compared to microglia of neonates born to NI mothers (Table 2). To confirm that LM^WT^ infection was responsible for the reduced expression of cell surface markers, we obtained microglia from NI mothers and infected them with LM^WT^, LM^∆LLO^ or LM^∆ActA^ mutants at MOI of 10:1 (bacteria: cell) and analysed cell surface markers by flow cytometry (Figure 3B). We confirmed significant reductions in CD11b^+^ (12% of positive cells), CD14^+^ (10% of positive cells) and CD86^+^ (2%-4% of positive cells) cell surface markers after microglial infection with LM^WT^, LM^∆LLO^ or LM^∆ActA^ mutants (Figure 3B). There were less prominent reductions in MHC-I^+^ after infection with LM^WT^ or LM^∆LLO^ and a greater reduction after infection with LM^∆ActA^ mutants (53% of positive cells). Confocal microscopy of purified microglia of P4 neonates born to NI mothers showed high staining of intracellular and membrane-bound CD11b (Figure 3B). However, cell surface staining of CD11b decreased significantly in microglia of P4 neonates born to LM^WT^-infected mothers, appearing mainly intracellular (white fluorescence) and co-localizing with GFP-LM^WT^ (green fluorescence) and F4/80 marker (red fluorescence) (Figure 3B). Co-localization of the three markers (white, green and red fluorescence) appeared as light yellow fluorescence (Figure 3B). We confirmed that LM^WT^ induced an apoptotic phenotype in microglia following the levels of the classical apoptotic marker, annexin V, by flow cytometry. Microglia of P4 neonates born to LM^WT^-infected mothers had 24.6% of cells with the apoptotic marker annexin V^+^, compared to only 2.1% in microglia of P4 neonates born to NI mothers (Table 2). To define the induction of apoptosis as early or late, we used a method that differentiates early and late apoptosis by fluorescence-activated cell sorting (FACS) as the double staining of cells with annexin V and 7-aminoactinomycin D (7-AAD) (late apoptosis) or single staining with annexin V (early apoptosis) in microglia of P4 neonates born to NI mothers and infected *in vitro* with different LM mutants. Microglial infection with LM^∆LLO^ or LM^WT^ induced early apoptosis in 9-12% of cells, annexin V^+^7-AAD^−^, (Figure 3C). LM^∆ActA^ infection of microglia failed to induce early apoptosis, since we only detected apoptosis in 1% of annexin V^+^7-AAD^−^ cells, which was similar to 0.9% of annexin V^+^7-AAD^−^ cells in control microglia (Figure 3C). We concluded that *actA* gene participated in LM induction of early apoptosis in microglia.

**Table 2:** Immune markers expression in microglia of mice neonatesa

	^b^Microglia-NI	Microglia-LM^WT^
**CD11b**	85 ± 0.7	8.2 ± 0.2
**F4/80**	35 ± 0.2	17.5 ± 0.5
**MHC-II**	53.7 ± 0.4	43.1 ± 0.5
**MHC-I**	98.1 ± 0.2	67 ± 0.2
**CD14**	20.5 ± 0.5	3.9 ± 0.1
**CD11c**	31 ± 0.08*	6.3 ± 0.2
**CD86**	27 ± 0.3	2 ± 0.08*
**Annexin V**	2.1 ± 0.2	24.6 ± 0.6

^a^Microglia isolated from P4 neonates born to either LM^WT^-infected (Microglia-LM^WT^) or ^b^NI (Microglia-NI) mothers were surface stained for the following markers: CD11b-FITC, F4/80-PE, MHC-II-APC, MHC-I-APC, CD14-PE and CD86-V450. Samples were acquired using a FACSCanto flow cytometer and percentages of positive cells for each antibody are shown. Results are expressed as the mean ± SD of triplicate experiments (*P* < 0.5), *(*P* < 0.1).

To establish whether LM induced pro- or anti-inflammatory apoptosis in microglia, we measured the pattern of cytokine release by microglia of neonates born to NI mothers, and infected them with LM^WT^, LM^∆LLO^ or LM^∆ActA^. Microglial infection with LM^WT^ induced high levels of TNF-α and IL-6 and significant levels of IL-10, but low levels of IL-1 or IFN (Table 3). However, microglial infection with LM^∆ActA^ failed to produce significant levels of TNF-α IL-6 or IL-10 and induced low levels of IL-1 and IFN. Microglial infection with LM^∆LLO^ only induced basal levels of most cytokines (Table 3). Similar cytokine patterns were observed in isolated microglia of P4 neonates born to LM^WT^-, LM^∆ActA^- or LM^∆LLO^-infected mothers (Table 3). We concluded that *actA* gene was relevant for LM^WT^-induced apoptosis in microglia, presenting a predominant Th2 cytokine pattern but with high levels of TNF-α.

**Table 3:** Anti-inflammatory pattern of microglial cells from P4 neonates

Activation pattern of Microgliaa	IL-6^a^	IL-10	TNF-α	IL-1	IFN-α
**CONTROL** (NI-Mo)	3.0 ± .01	1.2±0.1	6.0 ±0.1	1.0± 0.1	1.1±0.2
**LM^WT^** (NI-Mo)	70.0± 0.2	19.0±0.1	1385±0.6	5.0±0.1	5.5 ± 0.3
**LM^∆ActA^** (NI-Mo)	18.4 ± 0.6	5.4±0.1	23.0±0.9	22.0±0.5	18.2 ± 0.8
**LM^∆LLO^** (NI-Mo)	12.0 ± 0.3	3.2±0.1	18.0±0.7	1.0±0.1	8.5 ± 0.2
**LM^WT^**	106± 0.2	16.0±0.1	1315±0.6	6.0±0.1	9.1 ± 0.3
**LM^∆ActA^**	13.8 ± 0.6	5.6±0.1	21.0±0.9	20.0±0.5	25.0 ± 0.8
**LM^∆LLO^**	14.0 ± 0.3	3.0±0.1	17.0±0.7	8.0±0.1	8.0 ± 0.2

^a^Microglial cells from P4 neonates born to NI mothers (NI-Mo) and infected *in vitro* with different LM strains or born to LM^WT^, LM^∆ActA^ or LM^∆LLO^ infected mothers. Levels of pro-inflammatory cytokines were analysed using the CBA kit (Becton Dickinson) by flow cytometry. Microglial cells were infected for 20 min, incubated with 30 μg/mL gentamicin, and culture supernatants collected after 2 hour. All supernatants were filtered before storage at −80°C to remove bacteria from cytokine measurements. Results are expressed as the mean ± SD concentration (pg/mL) from three independent experiments (*P* ≤ 0.05).

### Characterization of the early transcriptional TNF-apoptotic program of microglia

**Figure 4:**
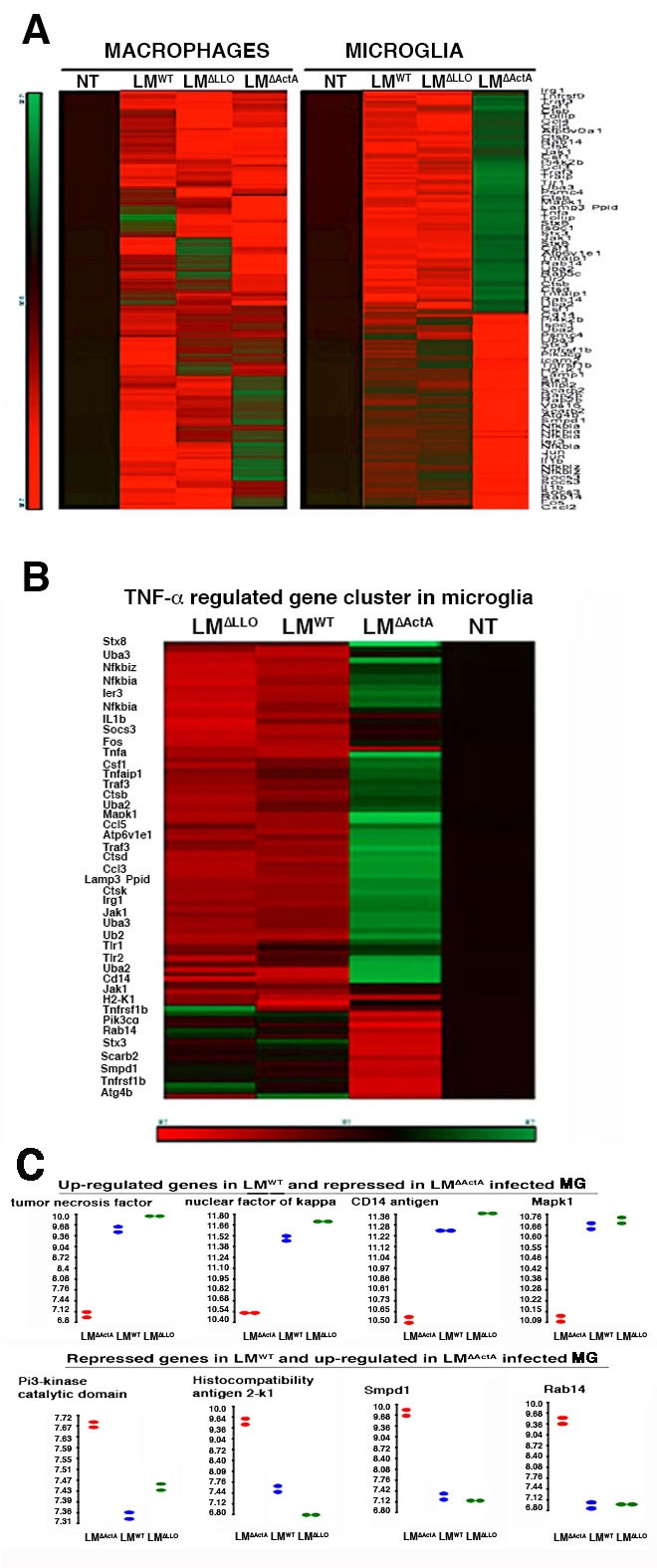
Characterization of early transcriptional program induced by LM **^WT^** in microglia. **A**. Heat map presentation of the 93 most differentially expressed genes of LM innate immunity cluster of bone-marrow macrophages (left) or microglia (right) infected with LM^WT^, LM^∆LLO^ or LM^∆ActA^ for 20 min or non-infected (NI). Coloured rows represent expression ratios from ≤ -2.0 Fold-change (FC)-repressed genes in green to ≥ 2.0 FC-induced genes in red. Black boxes correspond to non-differentially expressed genes. **B**. Microglia from P4 neonates born to NI mothers were infected with LM^WT^, LM^∆LLO^ or LM^∆ActA^ for 20 min or non-infected (NT), RNA was isolated and differential microarrays performed. Heat map presentation of the 32 most differentially expressed genes of LM innate immunity cluster. Coloured rows represent expression ratios from ≤ -2.0 Fold-change (FC)-repressed genes in green to ≥ 2.0 FC-induced genes in red. Black boxes correspond to non-differentially-expressed genes. **C**. Bioinformatic analysis of *actA* related genes overexpressed in LM^WT^-infected microglia (upper plots) that correspond to *tnf*, *nf-kb*, *cd14* and *mapk1* signalling genes. Genes related with *actA* and repressed in LM^WT^-infected microglial cells (lower plots) that correspond to *pi3Kcg*, *h-2k*, *smpd1* and *rab14* antigen processing trafficking.

We characterized the early apoptotic transcriptional program induced by LM^WT^ in microglia by differential expression of genes included on the Affymetrix GeneChip MOE430A2.0. We infected microglia *in vitro* with LM^WT^, LM^∆LLO^ or LM^∆ActA^ for 20 min and normalized all values using levels of NI cells. Of the 162 genes in the first selection, we applied innate immunity functional clustering and identified 93 genes that were differentially expressed in LM-infected cells (Figure 4A). We reduced the innate immunity functional cluster to 32 genes that were exclusively regulated by TNF-α (Figure 4B and Supplementary Table S1), which was the cytokine produced at highest levels after LM^WT^ infection of microglia (Table 3). LM^WT^-infected microglia presented two early expression programs regulated by *actA* gene: induction of a TNF-α death-regulated program, and repression of a pro-inflammatory expression pattern involved in macrophage activation and antigen processing. The TNF-α-induced death signature, included the TNF-α receptor-associated component genes, *tnf*, *traf3*, *tnfaip1* or *tnfrsf1b*; the TLR-associated genes, *tlr1*, *tlr2* and *cd14*; the NFKB signalling genes, *nfkbia* and *nfkbiz*; ant the mitogen-activated protein kinase (MAPK) gene, *mapk1* (Figures 4C and Supplementary Table S2); the early immune response gene, *ier3*; and two genes involved in ubiquitination, *uba2* and *uba3*. Other genes belonging to this signature were the early immune response gene, *irg1*, cytokine gene *il1b*, the chemokine genes *ccl3* and *ccl5*, the ion-pump ATPase regulatory gene *atp6v1e1*, and cathepsin genes *ctsb*, *ctsd* and *ctsk*, but with less intensity. The repressed pro-inflammatory signature in LM^WT^-infected microglia included the *pi3kcg* gene; the MHC I and pro-inflammatory *h2-k1* gene implicated in antigen presentation; the lysosomal acid sphingomyelinase genes, *smpd1* and *scarb2*, belonging to pro-inflammatory apoptotic and antigen-degradation routes; and the small GTPase component of cross-presentation signalling pathways, *rab14* gene (Figures 4C and Supplementary Figure S1). Other genes belonging to this repressed pro-inflammatory profile but with less intensity were the autophagy and inflammasome component, *atg4b*, and the SNARE component syntaxin 3, *stx3*. These results suggest a marked effect of LM^WT^ infection in the transcriptome of microglia and the induction of TNF-α-mediated death signalling controlled by *actA* gene, which was different from the transcriptional pattern that LM^wt^ induced in macrophages (Figure 4A).

### Effect of pregnancy vaccination with GNP-GAPDH1-22 nanovaccines formulated with the TLR2/TLR4 adjuvant, DIO-1, in neonatal listeriosis

**Figure 5:**
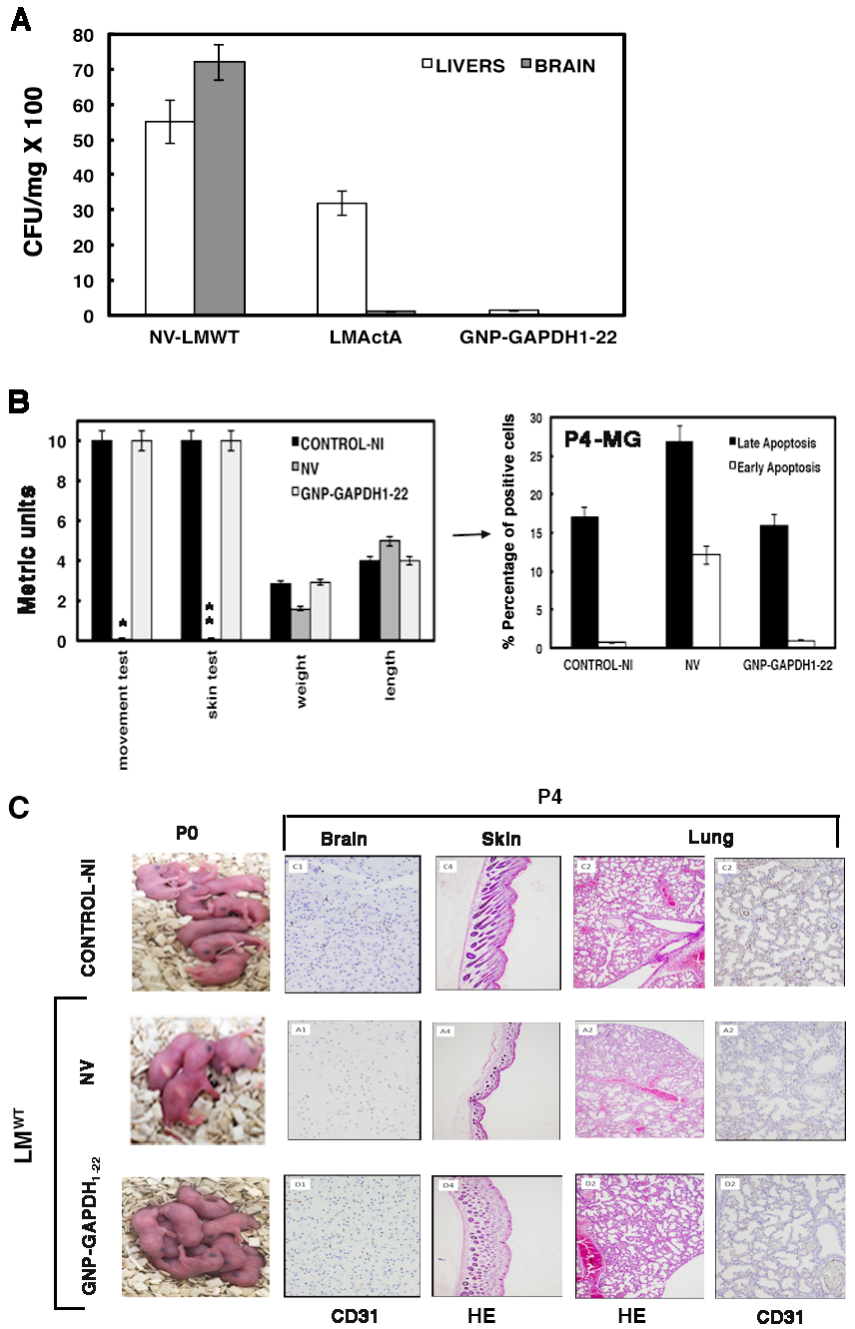
Effect of pregnancy vaccination with GNP-GADPH _1-22_ nanovaccines formulated with DIO-1 in neonatal listeriosis. **A**. P4 neonates born to NV (NV-LMWT), LM^∆ActA^-vaccinated (LM∆ActA) or GNP-GAPDH_1-22_-vaccinated mothers for 7 days. All groups were challenged with LM^WT^ for 3 days. P4 neonates and mothers were killed and livers and brains of P4 neonates were homogenized to determine number of viable bacteria in brain-heart infusion agar plates. Results are expressed as the mean ± SD CFU (*P* ≤ 0.05). **B**. Left plot corresponds to clinical tests performed in P4 neonates born to GNP-GAPDH_1-22_-vaccinated mothers challenged with LM^WT^ (GNP-GAPDH_1-22_), NV and challenged with LM^WT^ (NV) or control NI pregnant mothers (control-NI): movement test, skin test, length, weight and metrics were determined. Results are the mean ± SD of three different experiments. Bars, 0.1 ± 0.01 cm*, 0.5-1 melanoblasts/mm of skin**. Right plot corresponds to isolated microglia of P4 neonates born to control-NI, NV or GNP-GAPDH_1-22_ mothers, respectively and stained for annexin V-APC and 7-ADD. Results are expressed as the percentages of late apoptotic cells (black bars correspond to 7-ADD^+^ annexin V^+^) and the percentages of early apoptotic cells (white bars correspond to annexin V^+^ 7-ADD^−^ cells) (mean ± SD) (*P* ≤ 0.05). **C**. Left images correspond to P0 neonates born to control-NI, NV or GNP-GAPDH_1-22_ mothers; the two latter, challenged with LM^WT^. Right images show HE staining of skin and lungs from P4 neonates and immunohistochemical staining of CD31 marker in brain and lung sections.

Efficient vaccine vectors in listeriosis are expected to control pathogen dissemination to the CNS and liver, and release protective cytokines such as IL-12p40, while showing basal levels of acute anti-inflammatory cytokines such as IL-6 [10–12, 37–39]. In addition, vaccine vectors might ideally prevent microglial apoptosis that appears linked to brain-associated morbidity. We formulated GNP-GAPDH_1-22_ nanovaccines with DIO-1 as adjuvant that signals through TLR2 and TLR4, and induces IL-12 without causing excessive inflammation or IL-10 production [40]. Our purpose was to improve the spectrum of previous nanovaccine formulations with another adjuvant, Advax [39, 41] to prevent microglial dysfunction and brain disease in neonatal listeriosis. We vaccinated five pregnant mice at day 9 of gestation (E9), followed by challenge with LM^WT^ on day 16 of gestation (E16) for 3 days. We observed a lack of viable CFU/mL in brains and only 20-25 viable CFU/mL in the liver of P4 neonates born to GNP-GAPDH_1-22_-vaccinated mothers, indicating high efficacy to block LM^WT^ dissemination in neonates (Figure 5A). P4 neonates born to non-vaccinated (NV) mothers and infected with LM^∆ActA^ mutants (n = 5) showed 10-12 viable CFU/mL in brains but 3600 viable CFU/mL in the liver, indicating that these mutants disseminated in high numbers in the liver, severely crippled in their ability to invade the brain (Figure 5A). P4 neonates born to NV mothers and infected with LM^WT^ (n = 5) showed 7000 viable CFU/mL in the brain and 5800 viable CFU/mL in the liver, indicating high dissemination of bacteria in both organs (Figure 5A). The high efficiency of GNP-GAPDH_1-22_ nanovaccines to prevent dissemination in the brain and liver prompted us to focus on these vaccine vectors to prevent other listeriosis-associated morbidity. P4 neonates born to GNP-GAPDH_1-22_-vaccinated mothers and challenged with LM^WT^ showed normal ability to move (movement test), normal numbers of hair follicles (skin test), as well as normal weight and length compared to control neonates (Figure 5B). All these parameters were impaired in P4 neonates born to NV mothers and infected with LM^WT^ (Figure 5B). Isolated microglia of P4 neonates born to GNP-GAPDH_1-22_-vaccinated mothers also prevented early and late microglial apoptosis (Figure 5B) and shifted microglial cytokine production towards a Th1 pattern with high levels of IL-12p40 and high ratios of TNF-α/IL-6 (Table 4). We also detected high titres of anti-GAPDH_1-22_ antibodies in sera of GNP-GAPDH_1-22_-vaccinated mothers (GNP-GAPDH_1-22_ + LM^WT^) (Table 4), which is another indicator of vaccine efficiency in listeriosis [10, 39]. Microglia of P4 neonates born to NV and LM^WT^-infected mothers presented high levels of IL-6, IL-10 and TNF-α and lacked production of IL-12p40 (Table 4); a Th2 pattern similar to the cytokine profile reported in meningitis and encephalitic cases of listeriosis [4, 33, 35]. We also detected low titres of anti-GAPDH_1-22_ antibodies in sera of NV mothers and infected with LM^WT^ or LM^∆ActA^, suggesting low induction of specific immune responses (Table 4). Similarly, microglia of P4 neonates born to LM^∆ActA^-infected mothers failed to produce IL-12p40 (Table 4). GNP-GAPDH_1-22_ nanovaccination during pregnancy also prevented other manifestations of severe forms of listeriosis such as stillbirth or brain disease, since vaccinated mothers challenged with LM^WT^ gave birth to 8 ± 0.1 pups, similar to NV and NI mothers (Figure 5C) and showed normal cerebral cellularity and vascularization (Figure 5C). Histological examination and anti-CD31 immunostaining of P4 neonates born to GNP-GAPDH_1-22_-vaccinated mothers challenged with LM^WT^; indicated normal skin and mature lungs, spleen and stomach (Figure 5C) and corrected vascularization of these organs, indicating prevention of development retardation. GNP-GAPDH_1-22_ formulated with Advax, while showing listeriosis protection in brains (2-3 CFU/mL), failed to reduce liver LM^WT^ (400 CFU/ml) and produced anti-GAPDH_1-22_ antibodies, while produced high IL-10 levels (Table 4).

**Table 4:** Immune efficiency of GNP-GAPDH1-22 vaccination of pregnant mothers

CONDITION OF PREGNANT MICE^a^	Cytokine pattern of MG^a^					anti-GAPDH_1-22_ antibodies^b^
	TNF-α	IL-6^a^	IL-12p40	IL-10	TNF-α/IL-6	
**CONTROL-NI**	3.3± 0.1	3.2 ± .01	1.0±0.1	1.3 ±0.2	1.06 ± 0.1	0.15 ± 0.1
**NV-LM^WT^**	1190±0.9	105± 0.2	2.0±0.1	15.8 ±0.2	11.3 ± 0.7	0.41 ± 0.2
**NV-LM^∆ActA^**	25 ± 0.1	13 ± 0.6	1.9±0.1	5.7 ±0.1	1.9 ± 0.2	0.31 ± 0.1
**GNP-GAPDH_1-22_/DIO-1+ LM^WT^**	80 ± 0.1	3.4 ± 0.1	43±0.1	1.3 ±0.1	23.5 ± 0.3	1.85 ± 0.2
**GNP-GAPDH_1-22_/DIO-1 + NI**	20 ± 0.1	3.0 ± 0.1	13±0.1	1.3 ±0.1	6.6 ± 0.1	0.89 ± 0.2
**GNP-GAPDH_1-22_/Advax+ LM^WT^**	81 ± 0.1	7.9 ± 0.2	32 ± 0.1	12.1 ± 0.1	10.2 ± 0.2	0.45 ± 0.1
**GNP-GAPDH_1-22_/Advax+ NI**	18 ± 0-1	7.1 ± 0.1	10 ± 0.1	10-2 ± 0.2	2.5 ± 0.1	0.11 ± 0.1

^a^E16 pregnant mothers were NV and NI (CONTROL-NI), NV and infected with LM^WT^ (NV-LM^WT^) or LM^∆ActA^ (NV-LM^∆ActA^) or vaccinated with GNP-GAPDH_1–22_ nanovaccines (formulated with DIO-1 or Advax at 10 μg/mL) at E9 and challenged with LM^WT^ at E16 (GNP-GAPDH_1–22_ + LM^WT^). ^a^Pro-inflammatory pattern of microglial cells from P4 neonates born to mothers inoculated as above. Levels of pro-inflammatory cytokines were analysed by flow cytometry. Microglial cells were cultured *in vitro* for 2 h and culture supernatants collected. All supernatants were filtered before storage at −80°C to remove bacteria from cytokine measurements. Results are expressed as the mean ± SD concentration (pg/mL) from three independent experiments. ANOVA was applied to cytokine results (*P* < 0.05). ^b^Sera of mothers inoculated with the different LM strains were examined for anti-GAPDH_1–22_ antibodies by peptide ELISA. Results are presented as mean ± SD OD units from triplicate experiments (*P* < 0.05).

## DISCUSSION

Brain morbidities reported in neonatal listeriosis implied meningitis, brain abscesses, diffused skin lesions and rash, fever or lethargy [2, 9, 33–36]. We found that neonatal listeriosis in our health institutions, HUMV and HUD, represented 28% of all listeriosis cases but with high mortality rates of 50%, urging the development of safe vaccines to protect pregnant women at high risk of listeriosis and to prevent neonatal brain illness. The major morbidities we observed in our neonatal listeriosis cases were foetal death (patient HUD003 and second twin of patient HUD004) and severe meningitis in a premature neonate that was treated successfully with ampicillin (neonate HUD008 of patient HUD007) [9]. The incidence of listeriosis has increased in Europe since 2008 and especially in Northern Spain with two outbreaks, and current incidence rates of 1.71-1.86 cases per 100,000 inhabitants compared to 0.56 incidence rates before 2008 [1, 5, 9].

Therefore, vaccines can decrease incidence of listeriosis and increase immunocompetency in pregnant women. To prepare effective vaccines, we must identify immune biomarkers of good prognosis and the cells and mechanisms responsible for brain disease. Two immune biomarkers seem to be relevant in listeriosis, high titres of anti-GAPDH_1-22_ antibodies that indicate the ability of mothers with neonatal listeriosis to elicit potent LM-specific immune responses, and high Th1/Th2 ratios that prevent bacterial dissemination [9]. In this regard, all our patients developing neonatal listeriosis, had high titres of anti-GAPDH_1-22_ antibodies but low TNF-α/IL-6 ratios, confirming our efforts to formulate vaccines that increase Th1/Th2 ratios. Synthetic nanovaccines formulated with GNPs coupled to LM epitopes such as GNP-LLO_91-99_ or GNP-GAPDH_1-22_ have been tested successfully in systemic experimental listeriosis in adults [39, 41]. Since patients with listeriosis have failed to produce anti-LLO antibodies [9], we focused on GNP-GAPDH_1-22_ nanovaccines, selecting the adjuvant DIO-1 for the vaccine formulation, because it induces IL-12p40 and fails to produce IL-10 [40], avoiding disproportionate inflammation in the brain.

To explore the cells and mechanisms responsible for brain disease, we established a neonatal experimental listeriosis model by inoculating pregnant mice with pathogenic LM^WT^ at late pregnancy (E16). While not exactly similar, this experimental model of neonatal listeriosis mimics several human clinical symptoms of listeriosis [5, 9, 33–36, 42–44] and appears to retard normal foetal development (this study). In this regard, the threefold reduction in the number of neonates born to LM^WT^-infected mothers resembled spontaneous abortion (patient HUD003) or stillbirth (second born twin of patient HUD004), which were the most severe morbidities of neonatal listeriosis. Similarly, the enlarged heads, softer cranial covering and low number of neuronal bodies, are related with brain dysfunction and explained the lacked of coordinated movements, resembling severe brain diseases reported in neonates not treated with antibiotics [1, 2, 33]. Moreover, the lack of melanoblasts in the hair bulbs and a thinner epidermis with a delicate stratum corneum, are justified with a retard in the embryonic development at E16 stage induced by LM infection. At E16 stage, normally neural crest derivatives such as neurons, glial cells or melanoblasts start migration from the neural tube to brains or skin, respectively [45]. It is possible that LM infection causes impairment of melanoblast migration at this stage, since LM targets to melanocytes and induces apoptosis [46]. Moreover, other immature features, such as non-distended stomach, collapsed lung and reduction of blood vessels in the lungs and brain, are consistent with arterial ischaemic stroke and foetal bradycardia in early postnatal death [3, 36, 44]. These immaturities, together with the lack of spleen, retarded cerebellar formation and melanocyte migration to the skin in experimental neonatal listeriosis, which suggests severe delayed development, but also reduction of innate immune responses. Reduced innate immune responses explain dissemination of the pathogen in the liver and brain. Patients with neonatal listeriosis frequently presented with bacteraemia (HUD004 and HUD003), suggesting penetration of the blood-brain barrier and LM replication in the subarachnoid space, causing meningitis (patient HUD008) and cerebritis [33]. Neonatal meningitis caused by group B *Streptococcus* is associated with recruitment of microglia and induction of non-classical apoptosis [22, 25]. Similarly, microglia are the target cells in brains of mice with neonatal listeriosis, inducing a TNF-α-mediated transcriptional program of cell suicide that up-regulates early macrophage signaling via *tlr1*, *tlr2*, *tnf-α*, *nfkB*, *cd14* and *mapk1* genes with low induction of *ccl3* and *ccl5* chemokine genes, which suggests poor recruitment of monocytes to the brain compared to during systemic listeriosis [47]. This transcriptional program also down-regulates pro-inflammatory genes involved in macrophage activation and antigen presentation, such as *pi3kcg*, *h2-k1*, *smpd1*, *scarb2* and *rab14* genes. This transcriptional program is consistent with the cell surface apoptotic phenotype of microglia, CD11b^low^MHC-I^low^CD40^low^F4/80^high^MHC-II^high^AnnV^high^CD86^−^ [24] that appears to be regulated by the bacterial *actA* gene and not the classical *hly* gene that induces apoptosis in most phagocytes [27, 28]. This TNF-α-mediated apoptotic transcriptional program appears to be a mechanism to limit over-activation of innate immune responses, common to other bacteria inducing meningitis [22, 25] and consistent with high production of Th2 anti-inflammatory cytokines, IL-6 and IL-10, but low levels of Th1 cytokines such as IL-1β and IFN. Therefore, we consider this apoptotic pattern in neonatal mice as a TNF-IL-6 producing MG. The high numbers of CFUs detected in microglia of P4 neonates born to LM^WT^-infected mothers, verified the failure of microglial microbicidal mechanisms previously suggested *in vitro* [19], and therefore, microglial dysfunction. Microglial dysfunction in neonatal listeriosis explains brain disease such as motor impairment or lethargy.

Evaluation of two vaccine formulations, GNP-GAPDH_1-22_/DIO-1 and GNP-GAPDH_1-22_/Advax, in pregnant female mice before infection with LM, highlighted that DIO-1 adjuvant was superior to prevent neonatal listeriosis and avoid dissemination of the pathogen to the brain or liver, but also to avert brain disease associated with microglial failure. Neonates born to GNP-GAPDH_1-22_ nanovaccinated mothers showed normal numbers at birth with healthy conditions, coordinated movements, normal brain cellularity and vascularization, and no induction of apoptosis in microglia that produced high levels of IL-12p40 and TNF-α. These nanovaccines formulated with DIO-1, also induced high titres of anti-GAPDH_1-22_ antibodies in sera of vaccinated mothers, inducing LM-specific immunity and memory. We explained this broadened action of nanovaccines formulated with DIO-1 by implementing TLR2 signalling in different innate immune cells that elicit listeriosis-protective Th1 immune responses [40, 47–49]. The low production of anti-inflammatory cytokines IL-10 and IL-6 using DIO-1 as adjuvant prevented bacterial dissemination and induction of acute responses in the brain, preserving its integrity. Vaccine formulations with Advax or LM^∆ActA^ mutants failed to produce anti-GAPDH_1-22_ antibodies, reduced CFUs in the liver and induced IL-10 production, confirming GNP-GAPDH_1-22_ nanovaccine formulations with DIO-1 were superior. Nevertheless, Advax-formulated nanovaccines conferred significant listeriosis protection, while LM^∆ActA^ mutants did not.

We conclude that GNP-GAPDH_1-22_ nanovaccines formulated with a TLR2/4 targeted adjuvant, DIO-1, can be administered during pregnancy as they elicit Th1 protective immune responses and strong innate and specific immune responses. Their nanoscale formulation allows them to cross the placental barrier and induce a Th1 response in neonates that prevents brain and liver dissemination of the pathogen and establishment of listeriosis-associated diseases.

## MATERIALS AND METHODS

### Bacteria and peptides

We used *L. monocytogenes* strains EGD (ATCC) and 10403S (LM^WT^) and LM^∆LLO^ and LM^∆ActA^ mutants derived from 10403S strain (D.A. Portnoy, Berkley University, CA, USA), GFP-LM^WT^ and GFP-LM^∆ActA^ derived from EGD strain (M. Lecuit, Pasteur Institute, Paris, France) [38], and GFP-LM^∆LLO^ derived from 10403S strain (D.E. Higgins, Harvard Medical School, Boston, MA, USA). LLO_189-201_ and GAPDH_1-22_ peptides were synthesized at Centro Nacional de Biotecnología (CSIC, Madrid, Spain) followed by HPLC and mass spectrometry using a MALDI-TOF Reflex IV spectrometer. Peptide purity was ≥95% after HPLC.

### Animals

We used C57BL/6 mice from our animal facilities at the University of Cantabria at 8-12 weeks old. Three female and one male mice were mated and assessed for the appearance of vaginal plugs denoting first embryonic day of pregnancy.

### Patients and listeriosis cases

Listeriosis was confirmed in the foetus or stillborn or newborn infant according to the Commission Decision of 28/IV/2008 and classified as listeriosis at the Microbiology Department of Hospital Universitario Donostia (San Sebastian, Spain) during 2013-2015. In total, 13 human listeriosis episodes were detected in Gipuzkoa, which has an annual incidence of 1.86 cases per 100,000 inhabitants. Bacteria were recovered from blood in all cases. Description of patients and clinical manifestations is shown in Suuplementary Table *S2*. All patients participated in the study voluntarily and they gave signed informed consent at the time of physician consultation and received an information document about the study. Patients could revoke the informed consent at any time.

### Nanoparticles and adjuvants

To obtain GNPs carrying GAPDH peptide (GNP-GAPDH_1-22_), an aqueous solution of tetrachloroauric acid (Strem Chemicals, Newburyport, MA, USA) (0.025 M, 1 eq.) was added to a mixture of glucose (90%) and GAPDH peptide (10%) with thiol ending ligands (0.012 M, 6 eq.) in methanol/water/acetic acid (3:3:1). The detailed procedure has been reported previously [39]. The size distribution of the GNPs was evaluated from several transmission electron micrographs (JEM-2100F; Jeol, Tokyo, Japan), with an average diameter and number of gold atoms of 2.1 ± 0.5 nm. The presence of glucose and peptide ligands was confirmed by ^1^H NMR and the amount of GAPDH peptide on the GNPs was determined by quantitative NMR in a Bruker AVANCE 500 MHz spectrometer (Bruker, Billerica, MA, USA). GNPs (0.234 mg) were dispersed in 99.9% D_2_O (200 μL) and 80 μl of this solution was added to 40 μl 0.05% 3-(trimethylsilyl)propionic-2,2,3,3-d_4_ acid sodium salt solution in D_2_O as an internal standard. Peptide loading was 10.6 μg/0.234 mg GNPs and GNP-GAPDH_1-22_ and their stability in dendritic cells has been described previously [35]. DIO-1 is a TLR2/4 targeted molecule that can be used as an adjuvant [40] and Advax is an inulin formulated adjuvant [41].

### *In vivo* virulence of clinical LM isolates

C57BL/6 female mice were inoculated i.v. with 100 μL suspension of each clinical isolate in saline (5000 CFU/mice). At 72 h post-inoculation, mice were killed and spleens recovered and homogenized, and viable bacteria were determined on blood agar plates. Results are expressed as the mean ± SD of CFU/mL. All data were gathered in triplicate and we performed three independent experiments.

### *In vivo* model of neonatal listeriosis

Pregnant C57BL/6 female mice (n = 5) were inoculated intravenously (*i.v*) at 16 days of gestation (E16) via the lateral tail vain with 100 μL of a GFP-LM^WT^, GFP-LM^∆LLO^, GFP-LM^∆ActA^, LM^WT^, LM^∆LLO^ or LM^∆ActA^ bacterial suspensions in saline (3000 CFU/mL) (LM^WT^, LM^∆LLO^ or LM^∆ActA^ infected mothers) or inoculated with 100 μL of saline (non-infected (NI) mothers) (n = 5). All animals were examined daily. Three P4 neonates born to NI, LM^∆LLO^ or LM^∆ActA^ mothers and the two P4 survivors born to LM^WT^ infected mothers, were killed to obtain the cerebellum for preparation of mixed microglia and subsequent isolation of primary microglial cultures for CFU quantification, cell surface markers analysis by FACS, and cytokine quantification. LM^WT^-infected and NI mothers were bled for cytokine and anti-GAPDH_1-22_ antibody analysis and killed to obtain spleens and livers for CFU quantification and analysis of cell populations by FACS. Results were expressed as CFU/mL in spleens and livers and CFU/mL in microglial cultures ± SD of triplicates.

### Clinical tests in mice

Clinical tests were performed at P4 on all pups born to mothers infected or not with different bacterial strains. Metric tests of health conditions and units were as follows: weight (milligrams × 10), length (mm), movement assay placing pups in a 10-cm metric paper and recording the movement after 5 min (cm) and skin test, counting the number of black bulbs under a magnifying glass in 1 mm of skin, and skin was observed for general appearance as wrinkled or flattered. All results are the mean ± SD of three different experiments.

### Mixed microglial cell cultures and purified primary microglia

Microglial cultures and detailed procedures for obtaining mixed microglial cultures and purified primary microglia have been reported previously [19].

### Prenatal vaccination

Pregnant C57BL/6 female mice were vaccinated (n = 5) or not (n = 5) at day 9 of gestation (E9) via the lateral tail vain with GNP-GAPDH_1-22_ formulated with DIO-1 adjuvant (5 μg nanoparticles and 2 μg DIO-1). At 16 days of gestation (E16) all mice, were inoculated *i.v* with 100 μL of a LM^WT^ or LM^∆ActA^ bacterial suspension in saline (1 × 10^4^ CFU/mL) (NV-LM^WT^ or NV-LM^∆ActA^ infected mothers). All animals were examined daily. At E20 we detected 3 ± 0.5 pups born to LM^WT^ infected mothers, 4 ± 0.5 pups born to LM^∆ActA^ infected mothers and NV, while 8 ± 0.1 pups were born to control and GNP-GAPDH_1-22_ vaccinated mice, challenged or not with LM^WT^ (or LM^∆ActA^) (*P* ≤ 0.05). Results were the mean ± SD of three independent experiments. Four days after birth, P4 postnatal pups born to NV or GNP-GAPDH_1-22_-vaccinated and LM^WT^- (or LM^∆ActA^)-infected mothers were killed to quantify viable CFU/mL in whole brains and livers. Other P4 neonates were used for preparation of microglial cultures and measurement of cytokines and viable CFU/mL in these cells.

### Immunohistochemistry

Neonates were killed on day 4 after birth (P4) and were immersed in 4% formalin for cryopreservation of complete bodies. Most frequently affected organs (brain, skin, spleen, lungs and blood vessels) were resected, sectioned and fixed by immersion in 4% formaldehyde for 24 h. Organs were embedded in paraffin and cut at 3-μm thickness for histological analysis. Different sections (stained with hematoxylin and eosin; HE) of each organ were analysed by two independent pathologists. For immunohistochemical analysis, EnVision technology (Dako) was applied. Samples were boiled in target retrieval solution buffer as described previously [35]. Sections were incubated with ready-to-use primary monoclonal antibody (Dako) against CD31 to explore vascularized endothelia in neonatal organs. Antigen was visualized using biotinylated antibody and streptavidin conjugated with horseradish peroxidase (EnVision Mouse HRP; Dako). Diaminobenzidine (Dako) was used as the chromogen.

### Fluorescence label by confocal microscopy

Cells used for confocal microscopy were fixed in 3% paraformaldehyde. Fluorescence labelling and confocal microscopy were performed as previously described [10, 19].

### Flow cytometry analysis

Microglia purified from P4 neonates were cultured *in vitro* and cultured supernatants filtered and stored at −80°C before cytokine analysis using a CBA pro-inflammatory kit (BD Biosciences, NJ, USA) [10, 19]. Samples were analysed in triplicate and results are shown as the mean ± SD of three separate experiments.

Microglia were infected or not with different LM strains for 20 min (LM^WT^, LM^∆LLO^ or LM^∆ActA^). We isolated RNA and performed differential microarray analysis using the Affymetrix GeneChip MOE430A2.0 that evaluates 22,626 mouse genes [19]. The results of microarray analysis were deposited in NCBI Gene Expression Omnibus and accessible through GEO Series accession number GSE32505 (http://www.ncbi.nlm.nih.gov/geo/query/acc.cgi?acc=GSE32505). Results of the differential microarrays are expressed as signal log ratio (SLR). All final values were subtracted from basal controls values that corresponded to NI cells (detailed in Supplementary Information).

### ELISA for LLO189-201 and GAPDH1-22 peptides

We obtained sera from NV LM^WT^-infected, NV LM^∆ActA^-infected, NV NI, GNP-GAPDH_1-22_ vaccinated LM^WT^-infected and GNP-GAPDH_1-22_ vaccinated NI pregnant mice. Sera were also obtained from listeriosis patients. Peptide ELISA was performed as described previously [12, 39]. LLO_189-201_ and GAPDH_1-22_ peptides were prepared at 50 μg/mL in coating buffer (50 nM of Na_2_CO_3_ at pH 9.7) and used to coat Nunc MaxiSorp plates overnight at 4 °C. Plates were blocked with 1% BSA in PBS at room temperature for 30 min and mouse sera were diluted 1/200 in PBS/0.5% BSA and incubated for 1 h at room temperature. IgM was detected with a goat anti-mouse IgM conjugated with horseradish peroxidase or goat anti-juman IgM conjugated with horseradish peroxidase (0.8 μg/mL) and enzymatic reactions developed with tetramethyl benzidine in citric/acetate buffer and H_2_O_2_. Reactions were stopped with 50 μL H_2_SO_4_ (0.8 M) and absorbance was measured at 450 nm in the ELISA reader. Results were expressed as mean ± SD absorbance units from triplicate experiments.

### Statistical analysis

For statistical analysis, the Student's *t* test was applied. P≤0.05 was considered significant using GraphPad for graphic presentation. ANOVA was used for cytokine analysis (BD Biosciences, San Jose, CA).

### Ethics statement

This study was carried out in strict accordance with the recommendations in the Guide for the Care and Use of Laboratory Animals of the Spanish Ministry of Science, Research and Innovation. The Ethical Committee of Animal Experiments of the University of Cantabria approved this protocol (Permit Number: PI-01-17), which follows the Spanish legislation (RD 1201/2005). All surgery was performed under sodium pentobarbital anaesthesia and all efforts were made to minimize suffering. This study was also approved by the Ethical Committee of Clinical Research of Cantabria at Instituto de Investigación Marqués de Valdecilla (Santander, Spain), with the reference number 2014.228 (patients with listeriosis). All participants signed the Informed Consent documents and these documents are in the custody of physicians in accordance with Spanish Law (Ministry of Health).

## SUPPLEMENTARY MATERIALS FIGURE AND TABLES



## Abbreviations

CFU: colony forming units
GFP: green fluorescent protein
i.v.: intravenous
LM: *Listeria monocytogenes*
MG: microglia
TNF-α: tumour necrosis factor-α
