# Supplementary material for: GNP-GAPDH1-22 nanovaccines prevent neonatal listeriosis by blocking microglial apoptosis and bacterial dissemination
Source: Oncotarget. 2017 Jul 20;8(33):53916–34. doi: 10.18632/oncotarget.19405 (PMC5589551; doi:10.18632/oncotarget.19405)
Supplement: Supplementary file 1 [file oncotarget-08-53916-s001.pdf]

GNP-GAPDH$_{1-22}$ nanovaccines prevent neonatal listeriosis by blocking microglial apoptosis and bacterial dissemination

SUPPLEMENTARY MATERIALS

Supplemental Experimental Procedures

Reagents. FACS and confocal analysis used antibodies against CD14, CD11b, IA^d, IA^b and F4/80, which were conjugated with several fluorophores (BD Biosciences, NJ, USA), the neuron-specific mouse anti-beta III-tubulin antibody (TuJ-1) (Sigma, Saint Louis, MO, USA), the secondary antibody used was goat-anti-mouse IgG conjugated with Alexa 647 (Molecular Probes-Invitrogen, Carlsbad, CA, USA). Other FACS and confocal reagents were TRITC-phalloidin (Sigma) and DAPI (Molecular Probes, Eugene, OR, USA). Antibodies for western-blotting were mouse anti-Rab5a (4F11) (a gift from M. Zerial, Berlin, Germany), mouse anti-Jak1, mouse anti-Socs3, mouse anti-NFkB (Pharmingen, BD Biosciences, NJ, USA), rabbit anti-Scarb2/LIMP-2 (R & D Technologies, North Kingstown, RI), goat anti-Smpd1 (a gift from O. Uttermöhlen, Cologne, Germany), rabbit anti-Rab5c (a gift from M. Zerial, Berlin, Germany), mouse anti-TLR-2 (a gift from M. Fresno, Madrid, Spain) and rabbit anti-Pi3Kp110 (Santa Cruz Biotechnology, Santa Cruz, CA, USA). Other reagents used in the functional studies were murine pro-inflammatory CBA kit for cytokine measurements (BD Biosciences), murine recombinant macrophage colony-stimulating factor (M-CSF) (Sigma) and the culture media, Dulbecco's modified Eagle's medium, Iscove's modified Dulbecco's medium and fetal calf serum and supplements (Hyclone, Green Bay, WI, USA).

Mixed microglial cell cultures, purified primary microglia and macrophages. Mixed microglial cell cultures were performed as described [10]. Mixed microglia cell cultures were obtained from cerebellum at postnatal day 4 as described previously [46] from neonates born to GFP-LM^{WT}-infected or NI mothers. To visualize LM infection we fixed, immune stained and processed cultures for multichannel confocal microscopy. We purified primary microglia from mixed microglial cultures at day 7 by shaking at 200 rpm for 30 min. Cells in supernatants were re- in 24-well plates for confocal and FACS observations of the quality of microglial purification (microglial cells were 90% CD11b$^+$CD45$^+$ and 80–85% F4/80$^+$IA^{b+}), and supernatants were collected for cytokine analysis. Macrophages were derived from bone marrow from mice femurs and differentiated *in vitro* with M-CSF (20 ng/mL) for 7 days.

Fluorescence labelling, confocal microscopy examination and apoptosis. Cells used for immunocytochemistry were fixed in 3% paraformaldehyde. Actin was stained with TRITC–phalloidin. Activated microglia were labelled with F4/80-PE antibody. Quantification of FITC-labelled LM strains that co-localized with actin (TRITC–phalloidin) was performed as described previously [10]. Specimens were photographed using a Nikon A1R confocal microscope equipped. For apoptosis experiments, purified primary microglia (P4) were infected with LM^{WT} for 20 min and analysed for early and late apoptosis after staining with annexin V-APC and 7-AAD (BD Biosciences). Results are expressed as the percentages of late apoptotic cells (Late-A, Q2-2 area corresponding to double positive for 7-AAD$^+$annexin-V$^+$ cells) and the percentages of early apoptotic cells (Early-A, Q4-2 area corresponding to annexin V$^+$7-AAD$^-$ cells. Data are the mean ± SD of triplicates (P < 0.05) and statistical analysis performed with the Sigma Plot 8.0 program (BD Biosciences).

Transcriptional assays. Microglia and macrophages (10^6 cells/well) were cultured in six-well plates and infected or not with LM^{WT}, $LM^{\Delta LLO}$ or $LM^{\Delta ActA}$ (10:1 bacteria: cell ratio) for 20 min at 37°C. NI samples corresponding to basal level controls were cultured at the same cell density and treated as above. Total RNA was extracted from cells using the RNeasy total RNA isolation kit (P/N 74104; Qiagen, Hilden, Germany). The amount of total cellular RNA, integrity and quality were analysed and varied between 26 and 31 µg in all samples. RNA integrity was analyzed in 1% agarose gels (28S and 18S rRNA forms were observed as non-degraded in a 2:1 proportion). RNA quality was estimated by a close to 2.0 value of the ratio $A_{260/280}$ and the concentration was calculated with the assumption that 1 OD unit corresponded to 40 µg RNA measured at A_{260}. Differential microarray analysis was performed with the Affymetrix GeneChip MOE430A2.0 that evaluates 22,626 mouse genes with GCOS 1.3 Affymetrix software (Progenika Biopharma, Grifols, Derio, Bilbao, Spain). The fold changes in gene expression values were expressed as the SLR that corresponded to the $\log_2$ fold change (FC) of the previous version of Affymetrix software. Therefore, SLR $\geq$0.5 represented induced genes as they corresponded to values $\geq$2 FC; while SLR values ≤ -0.5 were depressed genes as they corresponded to values ≤ -2 FC. Gene ontology information (Affymetrix NetAffx Analysis Center; Progenika) and microarray analysis were deposited in NCBI Gene Expression Omnibus, accessible through GEO Series accession number GSE32505 (http://www.ncbi.nlm.nih.gov/geo/query/acc.cgi?acc=GSE32505).

Supplementary Table 1: Listeriosis patients: clinical manifestations and treatments.

Patients code[a]-Age (y)[c]<br>Period 2013-2015	Clinical manifestations	Type of infection	Listeriosis Treatment	Other Treatments
HUD001-57	Squamous cell glottis carcinoma	Bacteraemia	Ampicillin	Cisplatin
HUD002-36	Splenectomised autoimmune-hyperthyroidism	Bacteraemia	Ampicillin	Tirodril
HUD003-30	Pregnancy	Bacteraemia	NT	NT
HUD004-32	Pregnancy,<br>Cesarean-2nd twin lost	Bacteraemia	NT	NT
HUD005-74 (*deceased*)	Lung adenocarcinoma	Bacteraemia	Ampicillin	Cisplatin + holocranial radiotherapy
HUD006-60	Multiple myeloma IgGλ	Bacteraemia, meningitis	Ampicillin + gentamicin	Melphalan + radiotherapy + lenalidomide, velcade & dexamethasone
HUD007-30	Pregnancy, caesarean,	Corioamnionitis	NT	NT
HUD008-0	Premature neonate	Meningitis	Ampicillin	NT
HUD009-74	None	Meningitis	Ampicillin	Lisinopril
HUD010-90	Rhabdomyolysis	Bacteraemia	Ampicillin	NT
HUD011-54	None	Bacteraemia, blood diarrheal	Ampicillin	NT
HUD012-65	Type I hepatorenal syndrome	Peritonitis	Ampicillin	NT
HUD013-59	Hepatocarcinoma	Bacteriuria	Ampicillin	Surgery
HUMV001-89	Arteritis of Giant cells	Acute meningoencephalitis	Ampicillin	Prednisone
HUMV002-65	Hepatocellular carcinoma	Bacteraemia	Ampicillin	Ablation by microwaves
HUMV003-60	Guillian-Barre syndrome, sarcoidosis	Bacteraemia, hepatic abscess	Ampicillin+<br>Gentamicin	Prednisone
HUMV004-56	Cirrhosis-hepatic transplant	Brain abscess	Ampicillin	Prednisone +

				Micofenolate mofetil + Tacrolimus
HUMV005-49	Cutaneous primary lymphoma of Giant cells	Bacteraemia	Ampicillin+ Gentamicin	Rituximab + local radiotherapy
HUMV006-76 (*deceased*)	Prostate adenocarcinoma	Bacteraemia	ND	Taxocel
HUMV007-51	Squamous cell glottis carcinoma	Bacteraemia	Amoxicillin+ Clavulanic+ Ampicillin	Radiotherapy + cetuximab
HUMV008-28	Ulcerous colitis	Bacteraemia	Amoxicillin+ Clavulanic+ Ampicillin	Infliximab
HUMV009-54	Lung and bladder carcinoma	Bacteraemia	Levofloxacin	Cisplastin-ectoposide
HUMV010-57	Glomerulonephritis	Sepsis	Meropenem+ Ampicillin	Micofenolate mofetil + everolimus
HUMV011-51 *(deceased)*	Myeloblastic acute Leukemia	Bacteraemia	Amoxicillin+ Gentamicin	Azacitidin + cisplastin
HUMV012-84	Cutaneous Lupus	Bacteraemia	Amoxicillin+ Clavulanic+ Ampicillin	Prednisone
HUMV013-71	Glioblastoma multiforme	Bacteraemia	Augmentin	Temozolamide + Radiotherapy

[a]Clinical manifestations and treatments of listeriosis patients during August 2013–September 2015. Patients identified by internal codes. HUD: Hospital Universitario Donostia (Gipuzkoa), HUMV: Hospital Universitario Marqués de Valdecilla (Santander, Cantabria). [b]Age of the patient in years (y). NT, no treatment.

Supplementary Table S2: LM innate immunity cluster of microglial transcriptional response.

INFECTION STRATEGY[a]		Microglia-LM$^{\Delta ActA}$ vs. NI		Microglia-LM$^{\Delta LLO}$ vs. NI		Microglia-LM^{WT} vs. NI	
**GENE ONTOLOGY[b]**	**UniGene ID[c]**	***p*-value[d]**	**FC[e]**	***p*-value**	**FC**	***p*-value**	**FC**
**TNF-α regulated cluster:**							
Rab14	Mm.198264	0,0004	1,20	0	0	0	0
Stx3 (Syntaxin-3)	Mm.272264	3,29E-05	1,18	0	0	0,0013	-1,03
Stx8 (Syntaxin-8)	Mm.3973	0,0005	-1,19	0	0	0,024	-1,06
Atp6v1e1 (ATPase-H+/subunit E)	Mm.29045	6,85E-05	-1,25	0	0	0,023	-1,04
Uba2 (ubiquitin activating enzyme 2)	Mm.27560	0,0014	-1,14	0	0	0,049	1,12
Uba3 (ubiquitin activating enzyme 3)	Mm.277626	0,00015	-1,39	0	0	0,044	1,15
Atg4b (autophagy related 4B)	Mm.29087	0,0038	1,24	0,039	1,11	0	0
Lamp3 (lysosomal membrane pr3)	Mm.295252	0,0014	1,33	0	0	0,039	1,03
Scarb2 (lysosomal LIMP-2 prot)	Mm.297964	0,0001	1,22	0	0	0	0
Smpd1 (acid sphingomyelinase)	Mm.4628	0,0003	1,42	0	0	0	0
Ctsd (cathepsin-D)	Mm.231395	6,81E-05	-1,26	0	0	0	0
Ctsb (cathepsin-B)	Mm.236553	0,0016	-1,59	0	0	0	0
Ctsk (cathepsin-K)	Mm.272085	7,27E-09	-2,33	0,011	1,02	0,21	-1,01
Nfkbia (Nfkb light polipeptide al)	Mm.170515	0,0033	-1,20	0,036	1,77	8,4E-05	1,6
Nfkbiz (Nfkb light polipeptide z)	Mm.247272	0	0	5,1E-05	2,58	0,0026	2,01
MAPK-1 (MAP-kinase)	Mm.196581	0,0099	-1,36	0	0	0	0
Pi3kcg (PI3-kinase, catalytic pept)	Mm.101369	0,025	1,13	0	0	0,041	-1,11
Fos (FBJ osteosarcome oncogene)	Mm.246513	0,0052	1,76	2,3E-05	10,05	3,48E-05	8,04
H2-K1 (histocompatibility 2- K1)	Mm.422886	0,0001	1,17	0,021	-1,03	0	0
SOCS3	Mm.3468	0,0026	1,17	5,3E-05	2,51	9,91E-05	2,60
CSF1 (macrophage factor)	Mm.795	0,0270	-1,20	0,021	0	0	0
Tlr1 (Toll-like receptor 1)	Mm.273024	0,021	-1,30	0	-1,03	0	0
Tlr2 (Toll-like receptor 2)	Mm.87596	0,0063	-1,25	0	0	0	0
Ier3 (immediate early response 3)	Mm.25613	0,0008	-1,63	0,0001	2,22	0,0003	1,89
IRG1 (immunoresponsive gene 1)	Mm.4662	7,11E-05	-3,60	0	0	0	0
Traf3 (TNF receptor/assoc.factor 3)	Mm.27431	0,0002	-2,05	0	0	0	0

Tnfaip1 (TNF-α inducing prot. 1)	Mm.386774	0,0001	-1,21	0	0	0,018	-1,05
TNF-α (tumour necrosis factor)	Mm.1293	0,0002	-2,47	6,0E-05	3,4	0,0002	2,50
IL1b (interleukine 1-beta)	Mm.222830	0,0051	1,28	4,0E-05	2,45	0,0002	1,75
Ccl3 (chemokine CC ligand 3)	Mm.182574	1,74E-05	-1,9	0,015	1,11	0	0
Ccl5 (chemokine CC ligand 5)	Mm.284248	0,0001	-1,74	0,009	-1,19	0	0
**Housekeeping gene:**							
GAPDH	Mm.297	0	0	0	0	0	0

[a]Microglia (10^6 cells/well) were infected or not (NI) with LM^{WT}, $LM^{\Delta LLO}$ or $LM^{\Delta ActA}$ (ratio bacteria: cell of 10:1) for 20 min. NI samples were cultured at the same cell concentration. Total RNA was extracted from the cells and checked for quality (A_{260}/A_{280}) and concentration. [b]Gene ontology information was derived from Progenika (Affymetrix NetAffx Analysis Center). [c]Differential microarray data analysis was performed with the Affymetrix GeneChip MOE430A2.0 with GCOS 1.3, Affymetrix software that analyzed a total of 22,626 mouse annotated genes. Table shows the 50 differentially expressed genes of the LM innate immunity cluster. The FC of gene expression values is expressed as SLR relative to control samples, which corresponds to the log_2 of FC of previous Affymetrix software. A value >2 means the gene is up-regulated, and a value <2 means the gene is down-regulated. A value of 0 means no change.

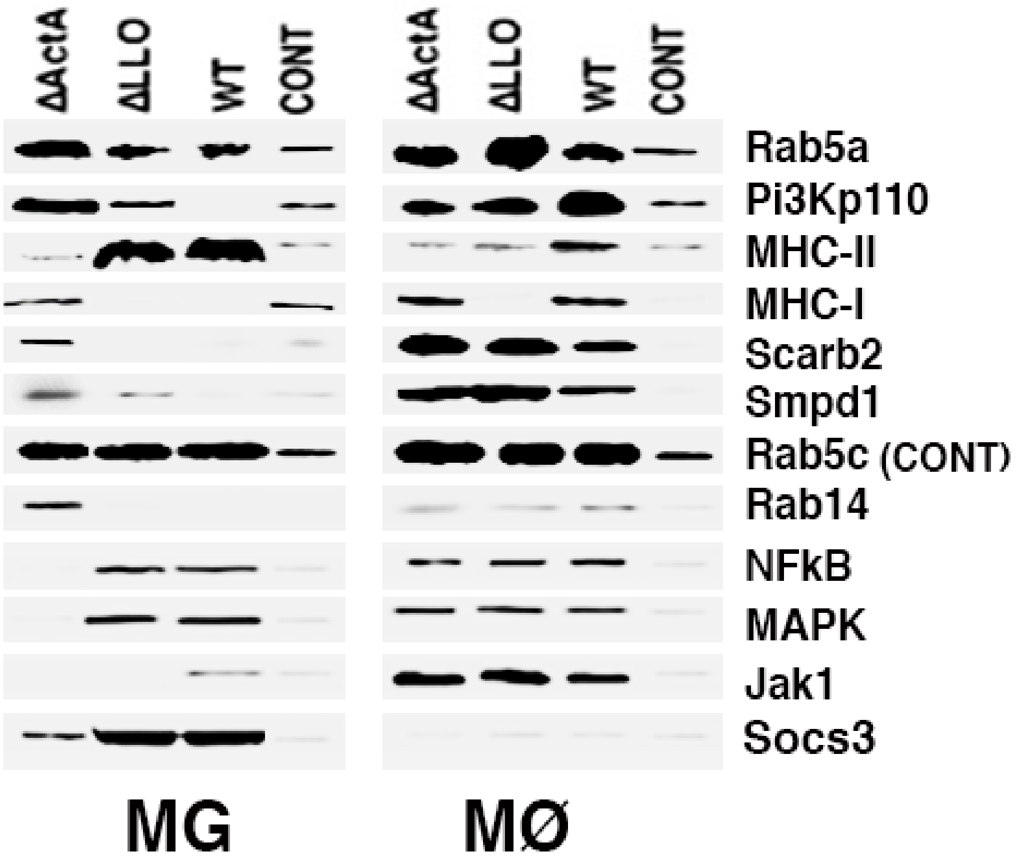

Supplementary Figure 1: Protein characterization of the TNF-mediated transcriptional program induced by LM in microglia. Western blots of 30 µg per lane of microglia or macrophage lysates after 20 min infection with the different LM strains or cells non-infected (CONT lanes) showed different relevant markers of immune components: Rab5a, Rab5c, MHC-I and MHC-II molecules, IFN regulated components as Jak1 and Socs3, innate immune and death signalling components: PI3Kp110, Smpd1, Scarb2 or death signalling. Blots were developed with specific antibodies.
